# Postoperative Ileus and Postoperative Gastrointestinal Tract Dysfunction: Pathogenic Mechanisms and Novel Treatment Strategies Beyond Colorectal Enhanced Recovery After Surgery Protocols

**DOI:** 10.3389/fphar.2020.583422

**Published:** 2020-11-24

**Authors:** Elvio Mazzotta, Egina Criseida Villalobos-Hernandez, Juan Fiorda-Diaz, Alan Harzman, Fievos L. Christofi

**Affiliations:** ^1^Department of Anesthesiology, The Ohio State University Wexner Medical Center, Columbus, OH, United States; ^2^Department of Surgery, The Ohio State University Wexner Medical Center, Columbus, OH, United States

**Keywords:** postoperative gastrointestinal tract dysfunction, postoperative ileus, colorectal enhanced recovery after surgery, gastrointestinal surgery, enteric glia, prokinetic agents, mechanosensation, 5HT4 receptor

## Abstract

Postoperative ileus (POI) and postoperative gastrointestinal tract dysfunction (POGD) are well-known complications affecting patients undergoing intestinal surgery. GI symptoms include nausea, vomiting, pain, abdominal distention, bloating, and constipation. These iatrogenic disorders are associated with extended hospitalizations, increased morbidity, and health care costs into the billions and current therapeutic strategies are limited. This is a narrative review focused on recent concepts in the pathogenesis of POI and POGD, pipeline drugs or approaches to treatment. Mechanisms, cellular targets and pathways implicated in the pathogenesis include gut surgical manipulation and surgical trauma, neuroinflammation, reactive enteric glia, macrophages, mast cells, monocytes, neutrophils and ICC’s. The precise interactions between immune, inflammatory, neural and glial cells are not well understood. Reactive enteric glial cells are an emerging therapeutic target that is under intense investigation for enteric neuropathies, GI dysmotility and POI. Our review emphasizes current therapeutic strategies, starting with the implementation of colorectal enhanced recovery after surgery protocols to protect against POI and POGD. However, despite colorectal enhanced recovery after surgery, it remains a significant medical problem and burden on the healthcare system. Over 100 pipeline drugs or treatments are listed in Clin.Trials.gov. These include 5HT_4_R agonists (Prucalopride and TAK 954), vagus nerve stimulation of the ENS—macrophage nAChR cholinergic pathway, acupuncture, herbal medications, peripheral acting opioid antagonists (Alvimopen, Methlnaltexone, Naldemedine), anti-bloating/flatulence drugs (Simethiocone), a ghreline prokinetic agonist (Ulimovelin), drinking coffee, and nicotine chewing gum. A better understanding of the pathogenic mechanisms for short and long-term outcomes is necessary before we can develop better prophylactic and treatment strategies.

## Introduction

Postoperative gastrointestinal dysfunction (POGD), commonly referred to as postoperative ileus (POI), is a widely known complication characterized by a transient impairment of gastrointestinal (GI) function after abdominal surgery. This clinical entity has been linked to a significant perioperative morbidity (e.g., enteral nutrition delay and patient discomfort) with subsequent financial burden due to extended hospitalization (Behm and Stollman, 2003; Wolthuis et al., 2016; Hedrick et al., 2018). In the United States, POI may increase hospital expenses up to 15% with an approximate annual cost of $1.46 billion (Goldstein et al., 2007).

Nausea, vomiting, abdominal tenderness and distention, absence of normal bowel sounds and/or delay in the passage of flatus and stool are some of the signs and symptoms associated with POI (Vather et al., 2013). However, the identification of other symptoms and risk factors may be significantly limited due to the ambiguous and heterogeneous definition of POI, making it even more difficult to estimate its overall incidence (Vather et al., 2013; Wolthuis et al., 2016). In 2018, the American Society for Enhanced Recovery After Surgery (ERAS) and Perioperative Joint Consensus considered forgoing the traditional definition of POI for a more functional definition and scoring system of POGD, to precisely describe the clinical manifestations of the GI Disorder (Hedrick et al., 2018). Therefore, a scoring system was proposed based on Intake, Feeling nauseated, Emesis, physical Exam, and Duration of symptoms (I-FEED), which defined three categories of postoperative GI functional impairment (Hedrick et al., 2018):

- Normal (I-FEED score 0–2): patients tolerate diet without bloating symptoms but may experience postoperative nausea and vomiting (PONV) within the first 24–48 postoperative hours.
- Postoperative GI Intolerance (POGI) (I-FEED score 3–5): these patients experience nausea, small-volume emesis, and bloating with or without bowel movements (stools or flatus) 48 h after surgery. However, most of them tolerate oral fluids and no nasogastric tube (NGT) is required.
- Postoperative GI Dysfunction (POGD) (I-FEED score >6): is the most severe level of impaired GI function. Patients develop painful abdominal distention with tympany, no bowel movements, nausea resistant to antiemetics and large-volume bilious emesis.

Currently, the mechanisms involved in the onset, duration and severity of POI and POGD remain unclear. Nevertheless, its association with spinal-intestinal sympathetic neural reflexes, sympathetic hyperactivity, inflammatory mediators, opioids use, electrolyte abnormalities and exacerbation by anesthetic or surgical techniques (e.g., size of the surgical incision and tissue manipulation), have all been extensively described as potential pathogenic mechanisms (Lobo et al., 2002; Luckey et al., 2003; Bauer and Boeckxstaens, 2004; Shah et al., 2011).

In addition, the occurrence and duration of POGD may also be determined by intestinal inflammation as measured in peritoneal fluid or surgical tissues, being longer periods of POI reported after open surgery when compared to laparoscopic minimally invasive procedures (Gomez-Pinilla et al., 2014; Vather et al., 2015).

This is a narrative review about POI and POGD and is not intended to provide a comprehensive description and understanding of the science related to the iatrogenic disease. The published scientific literature indexed in PubMed (Medline database), Scopus, LILACS and MEDSCAPE were searched for studies. The search focused on articles from 2015 to 2020, key words indexed in those articles and similar ones, as well as selected earlier publications. This review intends to focus on recent concepts of pathogenic mechanisms of POI and POGD, covering cellular targets and how current and pipeline treatments modulate these cellular mechanisms. An emerging cellular target of considerable interest is the “reactive enteric glial cell phenotype” in the pathogenic mechanism. CERAS guidelines for the management of POI and POGD, new therapeutic strategies and approaches to treatment in the neurogastroenterology field are discussed. Current Trials on POI and POGD listed in Clinical Trials.gov are briefly summarized in our review, with emphasis on cellular mechanisms.

## Pathogenic Cellular Mechanisms and Pathways Leading to Postoperative Ileus and Treatment Strategies

**FIGURE 1:**
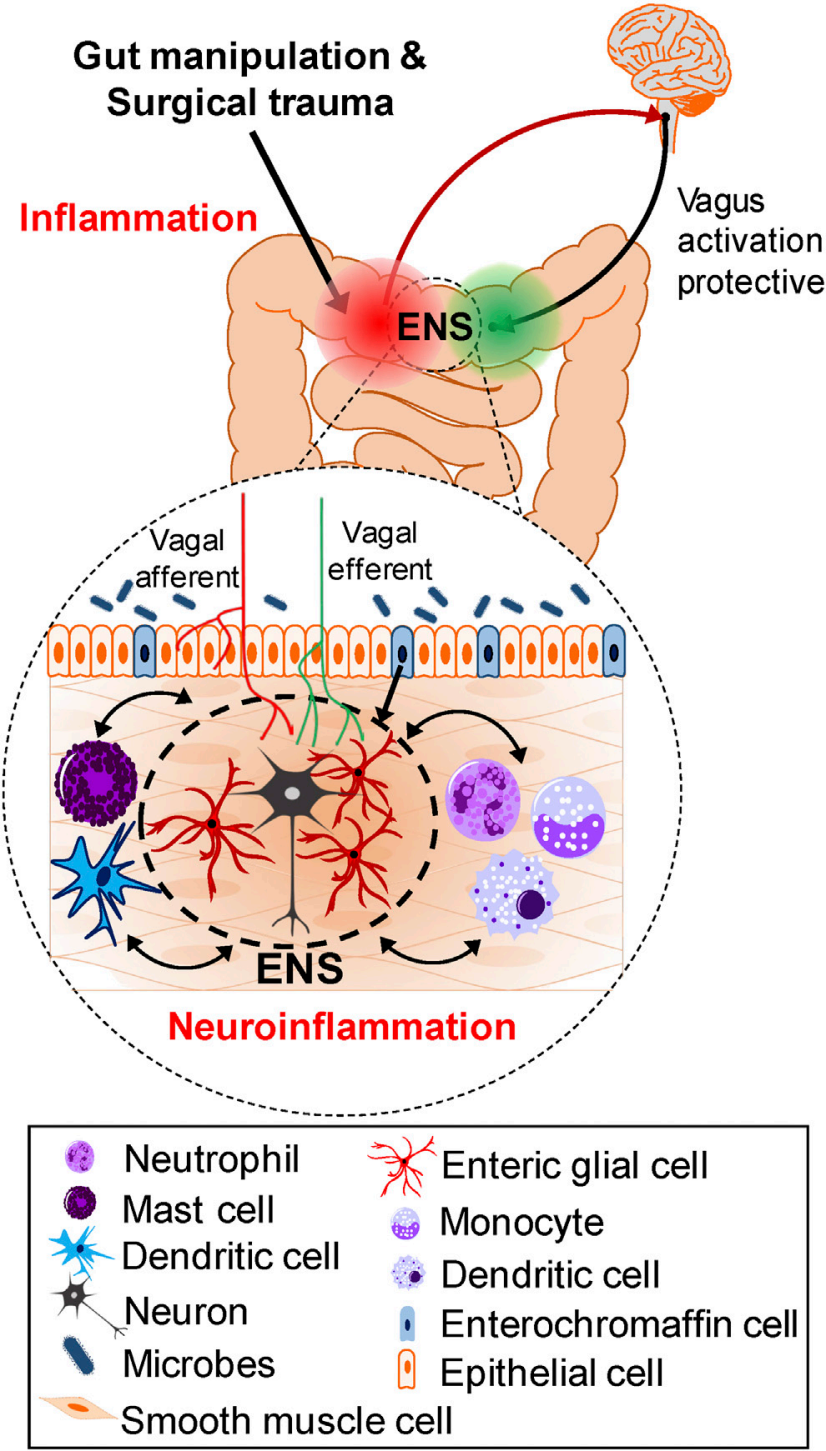
Cellular mechanisms and Pathways involved in the Pathogenesis of POI and POGD. Gut manipulation and surgical trauma triggers an inflammatory response in the muscularis externa. A variety of immune, inflammatory cells, enteric glia, neurons, smooth muscle cells, ICCs, enteroendocrine cells and microbes in the lumen of the gut, all contribute to the inflammatory response. Interactions in both directions exist between these cells in the intestinal wall but the precise trigger mechanisms activated for the cascade of events leading to POI are not known. In the context of inflammation, immune cell activation in coordination with reactive glia, promote monocyte and neutrophil infiltration, and all together produce a neuroinflammatory response leading to smooth muscle dysfunction and postoperative ileus. In contrast to chronic intestinal inflammation such as in IBD, after the surgery, in the absence of intestinal surgical manipulation, the ENS and intestinal motility eventually recover. The vagus—ENS—macrophage cholinergic (nAChR) pathway is a protective anti-inflammatory pathway, and vagus nerve stimulation can protect against development of POI and POGD.

This section will summarize pathogenic cellular mechanisms and signaling pathways leading to POI and highlight where in the inflammatory response following surgical intestinal handling treatments are intervening. Surgical trauma and gut manipulation trigger an inflammatory response in the muscularis externa involving a variety of immune, inflammatory cells (macrophages, dendritic cells, mast cells, monocytes, neutrophils), reactive enteric glia, neurons, smooth muscle cells, ICCs, enteroendocrince cells, epithelial cells, as well as the microbiome in the lumen of the gut. The resulting neuroinflammatory response leads to gastrointestinal dysfunction and postoperative ileus, associated with disturbances in motility, slower transit and constipation. Activation of the extrinsic nerve pathway via vagus nerve stimulation contributes to protective mechanisms. Figure 1 illustrates the cellular mechanisms implicated in the pathogenesis of POI and POGD—these are potential targets for treatment interventions.

The available evidence suggests it is likely that complex communication pathways exist between neurons, glia, immune cells, as well as other cells in the thickness of the gut wall, including ICC/fibroblast-like cells/smooth muscle cells that may contribute to the pathogenesis of POI and POGD. The precise interactions between different cells is not well understood. Findings from key recent studies on the role of various cell types will be summarized in this review. Muscularis macrophages are key immune cells in intestinal homeostasis and disease (Wehner and Engel, 2017; Stakenborg et al., 2019). Interactions between tissue macrophages and the enteric nervous system (ENS) are known to contribute to intestinal motility, serve as a protective mechanism during injury and infections, but they can also contribute to tissue damage in POI and other GI disorders. Extrinsic innervation modulates muscularis macrophages. Neuronal regulation of intestinal immune functions in health and disease was reviewed elsewhere (Fornai et al., 2018).

Enteric glial cells are involved in functional crosstalk with all other cells in the gut wall, including muscularis macrophages (Grubišić et al., 2020), although the consequences of all these communications remain largely unknown (Chow and Gulbransen, 2017; Fettucciari et al., 2017; Langness et al., 2017; Gulbransen and Christofi, 2018; Valès et al., 2018).

### Vagus—Intestinal Cholinergic (nAChR) Anti-Inflammatory Pathway, Vagus Nerve Stimulation, 5-HT_4_R Agonists, Enteral Nutrition

Goverse et al. (2016) recently reviewed the role of the intestinal cholinergic anti-inflammatory pathway involving the vagus nerve, the ENS and muscularis macrophages. Vagus nerve stimulation (VNS) has been shown to prevent POI in pre-clinical models by reducing activation of α7-nicotinic receptor (α7nAChR) positive muscularis macrophages and dampening surgery-induced gut inflammation (de Jonge et al., 2005; Cailotto et al., 2012; Matteoli et al., 2014; Stakenborg et al., 2017). In a pilot clinical study, VNS was associated with a significant reduction in interleukin 6 (IL-6) and IL-8 production in patients undergoing GI surgery (Stakenborg et al., 2017). The effect of VNS is likely mediated via enteric neurons to influence macrophages, since vagus nerve endings synapse with enteric neurons that are in close proximity to muscularis macrophages.

Enteric neurons dampen muscularis macrophage activation, and this effect is mimicked by prucalopride, the 5HT_4_ receptor (5HT_4_R) agonist (Stakenborg et al., 2019). Furthermore, similar to VNS, preoperative treatment with prucalopride protects against POI by preventing intestinal inflammation and shortening POI in both mice and humans. In humans, data was obtained from a randomized placebo-controlled pilot study of 42 patients. An earlier randomized clinical trial with prucalopride was also shown to reduce the duration of POI after elective gastrointestinal surgery, and it proved to be a safe and effective treatment without affecting post-operative complications (Gong et al., 2016). It could be concluded from these studies that preoperative administration of 5-HT_4_ agonists should be further evaluated as a prophylactic treatment of POI.

Another study with the 5HT_4_R agonist mosapride was shown to attenuate both macrophage and neutrophil recruitment into inflamed sites in experimental POI (Kimura et al., 2019). Recruitment of macrophage and neutrophils are regulated by different types of AChR, α7nAChR on muscularis macrophages and M2AChR (possibly in the ENS).

5-Hydroxytryptamine (5-HT) is one of the most important enteric modulators of the peristaltic reflex (McLean et al., 1997; Linan-Rico et al., 2016). The 5-HT_4_R agonist tegaserod was shown to be effective in treating constipation in a subset of patients diagnosed with constipation-predominant irritable bowel syndrome (C-IBS) (Prather et al., 2000; Evans et al., 2007). As a result, 5-HT_4_R agonists have emerged as a novel therapeutic alternative for patients experiencing GI motility disorders.

Recent evidence suggests that the 5HT_4_R agonist prucalopride reduces local tissue inflammation (Bouras et al., 1999; Gong et al., 2016; Stakenborg et al., 2019). Moreover, early studies in animals and healthy humans have shown that a new selective 5-HT_4_R agonist, TAK-954 (previously known as TD-8954) has a potent prokinetic effect. In a phase II clinical trial (NCT03281577), TAK-954 significantly improved gastric emptying when compared to metoclopramide (Beattie et al., 2011; Chapman et al., 2018). Currently, a phase II multi-center Clinical Trial with TAK-954 is underway on POI and POGD.

Abdominal surgery induces gastric ileus and activation of M1-like macrophages in gastric myenteric plexus is likely involved in the pathogenic mechanism since central vagal activation dampens postoperative gastric ileus and reduces intestinal inflammation (Yuan and Taché, 2017).

### TRPM2 in Macrophages

TRPM2 is a cation channel that is highly expressed in macrophages and other immune/inflammatory cells and regulates detrimental immune cell invasion in disease states (Haraguchi et al., 2012; Isami et al., 2013; Knowles et al., 2013; Gelderblom et al., 2014). Matsumoto et al. (2018) showed that activation of TRPM2 in resident muscularis macrophages induces release of chemokines and cytokines and in turn promotes infiltration of monocytes and neutrophils into the muscle to cause dysmotility. TRPM2 deficiency blocks or ameliorates these effects. Therefore, more studies are needed to explore the role of TRPM2 as a potential target in treating dysmotility due to POI.

### CXCL1 Release from Macrophages

The cytokine CXCL1 released from macrophages during intestinal surgical trauma was shown to suppress intestinal contractility. CXCL1 may provide another target for intervention to ameliorate POI and deserves further investigation (Docsa et al., 2020).

### CCR2-Dependent Monocyte-Derived Macrophages

In contrast to resident macrophages, CCR2-dependent monocyte-derived macrophages play a critical role in restoring intestinal homeostasis (Serhan and Savill, 2005; Shechter et al., 2009; Stoffels et al., 2009; Nahrendorf et al., 2010; Grainger et al., 2013; Zigmond et al., 2014) and this is also the case after surgical trauma in POI (Farro et al., 2017). GI transit recovery was delayed after gut manipulation in mice with defective CCR2-dependent monocyte migration to tissues (i.e., in Ccr2^−/−^ mice). Consistent with this, bone marrow reconstitution and treatment with macrophage colony stimulating factor 1 enhanced monocyte recruitment and differentiation of macrophages, and could restore GI transit in Ccr2^−/−^ mice by releasing anti-inflammatory cytokines. This raises the possibility that enhancing macrophage physiological repair functions is a potential treatment strategy for POI.

Monocyte-derived macrophages are the major source of IL-10 in POI. Leukocyte-derived interleukin-10 aggravates POI and in IL-10 deficiency, neutrophil extravasation into the postsurgical bowel wall is reduced and protects mice from developing POI (Stein et al., 2018).

### Mast Cells

Lipid rich enteral nutrition is a physiologic approach to activate the cholinergic vagal anti-inflammatory pathway by stimulating cholecystokinin receptors (Luyer et al., 2005). Early oral nutrition improves POI through the TRPA1/CCK1-R mediated mast cell-nerve axis. Activation of the TRPA1 pathway regulates CCK1-R to stabilize mast cells, but TRPA1 is not the target of the downstream CCK1-R pathway (Sun et al., 2020). In a randomized control trial, early enteral nutrition in patients undergoing major rectal surgery has been shown to reduce POI by improving recovery of gut motility, a reduction in the time to first defecation and length of hospital stay (Boelens et al., 2014). The contribution of mast cells to POI has been reviewed in experimental and clinical studies. Intestinal manipulation during surgery and mast cell degranulation releases pro-inflammatory mediators that can trigger formation of a localized infiltrate in the gut wall. The inflammation plays a significant role in POI by disrupting GI motility that also includes non-manipulated bowel segments (Peters et al., 2015). An earlier study provided proof of the concept that intestinal handling-induced mast cell activation and inflammation in human POI. Mast cell activation (tryptase release) and inflammation were determined in peritoneal lavage fluid in patients undergoing conventional and minimal invasive surgery. The study showed that intestinal handling triggers mast cell activation and inflammation associated with prolonged POI, that may in part explain faster recovery with minimal invasive surgery, although other cells and mechanisms are likely to be involved as well (The et al., 2008). The role of mast cells in functional GI disorders was reviewed by Wouters et al. (2016) and treatment with mast cell stabilizers offers a potential treatment strategy for IBS patients not responding to other therapies. Similar approaches deserve consideration in POI.

### Interstitial Cells of Cajal

Disruption of the pacemaker activity of interstitial cells of Cajal (ICC) via a nitric oxide (NO) pathway contributes to POI (Kaji et al., 2018). Administration of aminoguanidine, an inducible NO synthase inhibitor, suppressed the disruption of the ICC networks in POI. Acupuncture protects ICC’s in part by regulating miR-222 in a rat model of POI (Deng et al., 2019), although the underlying mechanisms remain unclear.

The effects of acupuncture on POI were also assessed in patients after colorectal resection and in colocolic anastomosis mice. Acupuncture was shown to inhibit macrophage activation, IL-6 release and miR-19a up regulation (an inflammation-related miRNA), while promoting anol restoration and KIT in ICCs. Acupuncture also reduced high serum miR-19a level in patients with colorectal surgery. In this study, acupuncture ameliorates POI via the IL-6-miR-19a-KIT Axis to protect ICC’s (Deng et al., 2017).

### Role of Endogenous Gases Nitric Oxide, Carbon Monoxide, and Hydrogen Sulfide in Postoperative Ileus

The endogenous gases NO, carbón monoxide (CO), and hydrogen sulfide (H_2_S) play a role in POI. The possible involvement of NO in the pathogenesis of POI was described many years ago (Moojen et al., 1999). Carbon monoxide treatment was shown to ameliorate POI in mice (Moore et al., 2003; Nakao et al., 2006). More recent studies have shown that H_2_S is involved in cellular signaling and cytoprotection of the colonic mucosa and other organ systems (Calvert et al., 2010; Matsunami et al., 2012; Kimura, 2013). For example, H_2_S releasing nonsteroidal anti-inflammatory drugs (NSAIDs) protect the mucosa from ulceration (Wallace, 2007; Ekundi-Valentim et al., 2013; Magierowski et al., 2017). Release of H_2_S contributes to the anti-inflammatory effects of H_2_S-NSAIDs, by reducing leukocyte infiltration, COX-2 activity and IL-1β expression. A recent study in mice showed that the H_2_S-releasing naproxen derivative ATB-346 and the slow releasing H_2_S donor GYY4137 were effective in reducing intestinal inflammation and restoring transit in postoperative ileus (Van Dingenen et al., 2019), suggesting that targeting the H_2_S pathway in the gut is a potential target for developing a prophylactic treatment for POI. A systematic review and meta-analysis revealed that NSAIDs reduce the time to recovery of gut function after elective colorectal surgery (Milne et al., 2018). Clinical trials with H_2_S-releasing NSAIDs seem feasible and safe since ATB-346 has already been tested in phase 1 and phase 2 studies in patients with osteoarthritis (Wallace et al., 2018). The underlying mechanisms by which H_2_S releasing compounds exert their beneficial effects in POI remain unknown.

### Acupuncture, Electroacupuncture and Nucleus of the Solitary Tract Neurons

In China, acupuncture has been traditionally used as an alternative treatment of GI disorders (Takahashi, 2006). Even though several studies have determined the effectiveness of acupuncture in the prophylaxis against POI after colorectal surgery, current clinical evidence remains inconclusive (Meng et al., 2010; Deng et al., 2013; Ng et al., 2013; Zhang et al., 2014). Additional randomized controlled studies are necessary to prove or disprove its effectiveness.

Electroacupuncture (EA) is a modern way of delivering acupuncture used widely in various GI diseases around the world. EA administered at ST36 shortened the recovery time of GI and colonic transit and increased gastric emptying. The beneficial effect of EA on POI was thought to be mediated by exciting neurons in the nucleus of the solitary tract (NTS) and activating the vagus efferent nerve pathway to improve GI tract transit, but not by activating the cholinergic anti-inflammatory pathway (Fang et al., 2017).

### Enterochromaffin Cells

Transient receptor potential ankyrin 1 (TRPA1) agonists improve intestinal transit in a mouse model of POI (Tsuchiya et al., 2016). TRPA1 receptors on enterochromaffin cells are a potential cellular target for the action of TRPA1 agonists. Intraluminal TRPA1 stimulation is suggested to be a potential therapeutic strategy for POI and GI motility disorders.

### Mesothelial Cells

Inflammation in intestinal mesothelial cells in the abdominal cavity is an important pathogenic mechanism in clinical conditions such as POI and peritonitis. The anti-inflammatory pathway regulated via α7nAChR in rat intestinal mesothelial cells may also involve enteric nerves (Mihara et al., 2017).

### Microbiome

Small bowel mucosal antimicrobial defense is disturbed in a gut manipulation mouse model of POI and it is accompanied by bacterial overgrowth and translocation. IL1R activation is involved in gene expression of mucosal antimicrobial peptides that serves to protect the epithelium from an increasing microbial challenge (Stein et al., 2018).

### HuR/p38/MK2 Signaling Pathway

In experimental studies in a mouse model of intraperitoneal transduction of HuR-RNAi lentivirus, suppression of HuR gene expression in mouse POI was shown to cause a significant reduction in inflammation (in infiltration of inflammatory cells, expression of pro-inflammatory genes, and reduction in serum cytokines) via the p38/MK2 signaling pathway (Xiong et al., 2017). The study did not evaluate the impact of Lentivirus-mediated HuR RNA interference on restoring normal GI transit in the POI model. Data suggest that HuR is a potential candidate drug target for the mitigation of POI, and further studies are necessary to prove this.

## Future Perspectives: Targeting Enteric Glia

Despite the implementation of CERAS protocols, POI and POGD remain a significant medical problem and burden on the healthcare system. A better understanding of the pathogenic mechanisms of POI and POGD in both experimental models and in the clinical setting are desperately needed. A cellular target of growing interest in the field of neurogastroenterology and motility is the enteric glial cell and in particular in GI disorders. The following section is focused on the role of glia as a therapeutic target in POI and POGD.

Enteric neuropathies are a hallmark of GI Diseases and Disorders. A growing body of evidence supports the concept that enteric glia are involved in the pathogenesis (Ochoa-Cortes et al., 2016; Gulbransen and Christofi, 2018) and “reactive glia” contribute to neuroinflammation and abnormal motility. There is great interest in enteric glia in the field of neurogastroenterology and motility—they are implicated in GI diseases and disorders including IBS, IBD, postoperative ileus, chronic morphine-induced constipation, and idiopathic constipation (Ochoa-Cortes et al., 2016; Gulbransen and Christofi, 2018). Glial Ca^2+^ waves are required for normal motility, and disruption of these waves disrupts motility. Enteric glia and neurons contribute to enteric neuropathies underlying these disorders (Ochoa-Cortes et al., 2016; Chow and Gulbransen, 2017; Gulbransen and Christofi, 2018).

### Pathogenesis of Postoperative Ileus and Postoperative Gastrointestinal Tract Dysfunction-Reactive Glial Phenotype

**FIGURE 2:**
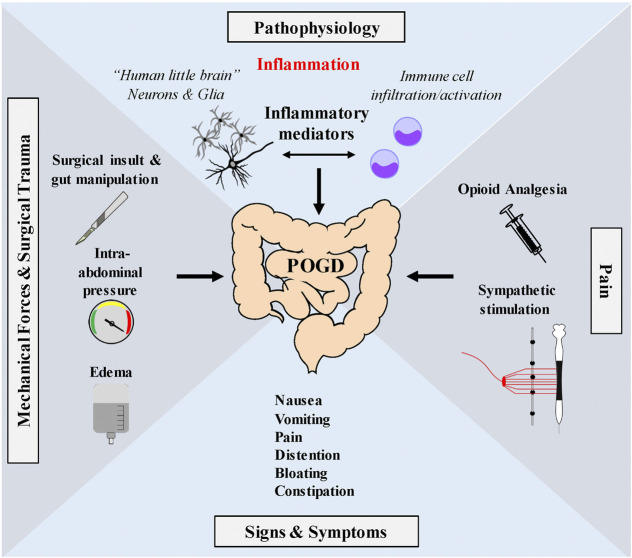
Working hypothesis of proposed glial pathogenic mechanism of postoperative ileus—Enteric glia are very sensitive to mechanical stimulation and mechanical forces generated during peristalsis. Touch, stretch, shear stress, pressure, compression, membrane perturbations and centrifugal forces all operate during peristalsis. Mechanosensation is a normal function of enteric glia in the modulation of motility through interactions with the ENS. Abnormal mechanical forces encountered during GI surgery such as gut manipulation, surgical insult, fluid edema or high pressure pneumoperitoneum encountered in minimal invasive laparoscopic surgery, may activate enteric glia (and immune cells) in the muscularis externa contributing to the induction of a reactive enteric glial cell phenotype. Reactive glia in coordination with immune cells release pro-inflammatory mediators that disrupt the ENS to cause GI dysmotility associated with POI and POGD. Signs and symptoms include nausea, vomiting, Pain distention, bloating and constipation. Pain pathways also activate the sympathetic nervous system which has inhibitory effects on GI motility. Additionally, opioids, commonly used during the perioperative period to treat pain, activate peripheral µ opioid receptors in the ENS and further depress peristalsis.

A working hypothesis of the pathogenic mechanism of POI and POGD is illustrated in Figure 2. Enteric glial cells modulate neural circuit activity in the enteric nervous system (ENS) or gut “little brain” and are required for normal motility. Disruption of glial cell activity leads to abnormal motility. Enteric glia is activated by mechanical forces encountered during peristalsis that are generated by coordinated movements of the gut. Glial activation is involved in ongoing fine-tune modulation of motility through the ENS. Abnormal mechanical forces on the gut and its mesentery are encountered during intestinal surgery, which are believed to cause a “reactive glial” phenotype. Reactive glia contributes to neuroinflammation and abnormal motility associated with POI.

Gut surgical manipulation and trauma, holding the bowel in place with a self-retaining retractor throughout the case, exerting pressure on the segment or squeezing and stretching the gut can activate glia and convert them to a pathogenic state referred to as “a reactive glial phenotype” leading to POI and POGD. Edema and high-pressure pneumoperitoneum (high PNP) resulting from inflating the peritoneal cavity during laparoscopic surgery can also activate glia. There is more finger manipulation of the bowels with open surgery, but laparoscopic and robotic instruments use higher pressure over smaller areas than one’s fingers. In laparoscopic operations, patient positioning and gravity are used for mass movement, but abdominal contents are exposed to increased serosa pressure from carbon dioxide insufflation. In addition to such high PNP, edema from intravenous fluids causes swelling and stretch of glia to activate them (Cooke et al., 2003; Christofi et al., 2004; Alcaino et al., 2017). Mechanogated channels are activated by such abnormal mechanical stimulation of the bowels during intestinal surgery—The types of channels involved are under investigation, but so far, the type of channel(s) linked to glial mechanosensation remain elusive. Candidate channels include various transient receptor potential channels, Piezo 1, 2 channels, connexin hemichannels, pannexin channels, P2X7 channels and Aquaporin channels (Kirischuk, 2008; Alcaino et al., 2017; Suchyna, 2017; Wang et al., 2017). A better understanding of these channels in enteric glia is important in developing better strategies to prevent POI and POGD.

### High Vs. Low-Pressure Pneumoperitoneum

Despite lower incidence of POI with a minimal invasive approach compared with open surgery (laparotomy) (Behm and Stollman, 2003; Bragg et al., 2015) and improvements seen with the implementation of CERAS protocols, this technique requires carbon dioxide (CO_2_) and higher abdominal pressure in order to enhance laparoscopic visualization for surgery. These factors also contribute to POI and POGD and it can adversely affect the patient’s homeostasis, leading to cardiovascular and respiratory systems changes, as well as a decrease in perfusion of abdominal organs (Bragg et al., 2015; Schietroma et al., 2016). High-pressure pneumoperitoneum (PNP), may cause systemic inflammation and affect the immune response in the early postoperative period (Schietroma et al., 2013; Schietroma et al., 2016; Vasdev et al., 2018; Rohloff et al., 2019). In order to overcome such adverse effects, low CO_2_ PNP pressure could potentially be used to reduce the risk of POI and POGD by reducing postoperative inflammatory response (circulating levels of inflammatory mediators or intestinal inflammation) and immune suppression.

It is hypothesized that the use of low PNP would reduce intestinal inflammation, protect against smooth muscle dysfunction, POI and POGD. To date, no clinical trials have tested whether low-pressure pneumoperitoneum is protective against intestinal and systemic inflammation, POI and POGD. Our ongoing research has shown that intestinal glial cells are very sensitive to physiologic mechanical forces such as those occurring during peristalsis or excessive forces such as occur during surgical manipulation. During laparoscopic surgery, excessive external mechanical forces generated by high-pressure pneumoperitoneum may overcome the autoregulation capacity of the intestinal glia, and the constant increased intra-abdominal pressure (IAP) which result in bowel compression would induce a reactive glial phenotype. Therefore, one could expect that the reactive glia phenotype may be responsible for some of the common postoperative complications in patients undergoing laparoscopic abdominal surgery (Liñán-Rico et al., 2016; Gulbransen and Christofi, 2018). In a recent retrospective study, lower pneumoperitoneum pressures were associated with a reduced incidence of POI and LOS in 400 patients undergoing robotic-assisted radical prostatectomy (Rohloff et al., 2019). Similarly, a decreased postoperative inflammatory response and attenuation of postoperative immunosuppression/human leukocyte antigen-DR receptor expression were reported in patients undergoing laparoscopic Nissen fundoplication (LNF) with low pneumoperitoneum pressures (Schietroma et al., 2013).

**FIGURE 3:**
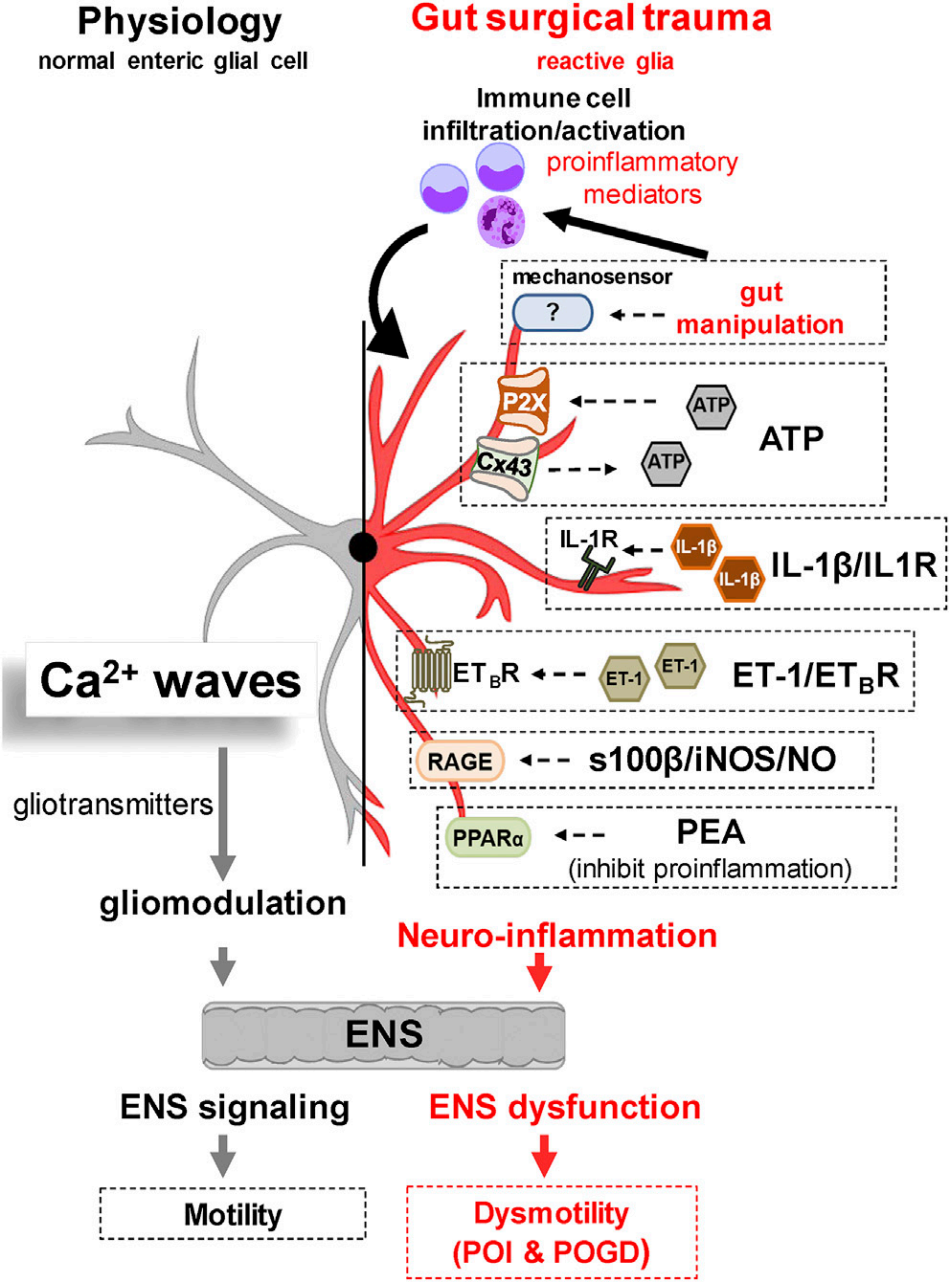
Working hypothesis of Glial Molecular Mechanisms Implicated in the Pathogenesis of POI. Under normal physiological conditions, glial cells modulate motility by interacting with neural-motor components of the gut. Glia communicate with each other and the ENS via Ca^2+^ waves and release of gliotransmitters. Recent evidence has revealed a number of potential glial targets implicated in the pathogenesis of POI and POGD. Gut surgical trauma and manipulation induces a reactive enteric glial phenotype that contributes to the overall neuroinflammation and GI dysmotility. Experimental evidence in reactive glia suggests that a variety of glial molecular signaling mechanisms may be operating in POI. These include 1) abnormal mechanosensation, 2) purinergic pathways via ATP, 3) the IL1β/IL1R Signaling Pathway, 4) the ET-1/ETBR signalling pathway, 5) the s100β-RAGE/iNOS/NO signaling pathway, and 6) a PPARα signaling pathway targeted by PEA to inhibit inflammation.

Reactive glia together with other cells in the gut muscularis externa (i.e., immune cell infiltration, neutrophils, monocytes, resident macrophages and smooth muscle cells) produce and release pro-inflammatory mediators and activate pathogenic mechanisms to induce POI and POGD. Diverse pathways and mechanisms in reactive glia such as mechanogated channels, IL-1β signaling, growth factors, s100β signaling, ATP signaling, Cx43 hemichannels, PPAR alpha receptors, and chemical messengers like nitric oxide (NO) can alter ENS function, induce enteric neuropathy, and cause POI. These are potential novel glial targets for future drug development that have been described in a recent review in IBD (2016) (Ochoa-Cortes et al., 2016) and a commentary in Gastroenterology by Gulbransen and Christofi (2018). Therefore, they will only be given a brief mention here. Potential pathogenic mechanisms targeted in preclinical studies for POI are shown in Figure 3.

### Edema

Edema alone can decrease intestinal contractile activity (Uray et al., 2006; Chu et al., 2012). During abdominal surgery and surgical trauma, edema results in increased stretch of intestinal smooth muscle cells that was shown to down-regulate MLC phosphorylation (Chu et al., 2013). Therefore edema in the gut wall and increased intestinal wall stress (Cox et al., 2008) can also cause smooth muscle dysfunction after intestinal surgical trauma.

## Experimental Targets for Therapy on Enteric Glia

### IL-1β/IL1R Signaling in Glia

Interleukin-1β activation of interleukin-1 receptor (IL-1R) in enteric glia is a potential pathogenic mechanism in experimental postoperative ileus induced by gut surgical manipulation and trauma. In pre-clinical studies, Sven Wehner’s group identified IL-1β and IL-1R signaling in enteric glia as a potential contributing mechanism to GI motility disruption and POI. These findings were supported by the fact that the IL-1β receptor antagonist “anakinra,” was effective in reducing inflammation and POI. Antagonist drugs at IL-1R such as anakinra are in clinical use for rheumatoid arthritis and their therapeutic benefit could be tested in POI and POGD (Stoffels et al., 2014). A recent study showed that the “absent in melanoma 2” (AIM2) inflammasome-derived IL-1β induces POI in mice. IL-1β production depends on AIM2 inflammasome formation and the microbiome. Targeting this pathway might also be a promising target to prevent POI in surgical patients (Hupa et al., 2019).

### s100β Protein in Glia

Glial s100β protein is a marker of enteric glia but is also involved in inflammation caused by pathogenic bacteria such as infection with *Clostridium difficile* and it involved in UC and celiac diseases (Esposito et al., 2007). Bacterial products activate the glial toll-like receptor—4 via the s100β-RAGE/iNOS/NO signaling pathway via nuclear transcription factor nfkB. This pathway is involved in ENS neural dysfunction. RAGE inhibitors or drugs that interfere with different components of the pathway, such as a NOS inhibitor L-NMMA that demonstrated efficacy in a clinical trial could be tested in POI and POGD (Galasko et al., 2014).

### PPARα Receptors

Targeting PPARα receptors with palmitoylethanolamide (PEA), a receptor agonist, can inhibit pro-inflammatory responses in reactive enteric glia in pre-clinical IBD models. Testing PEA in a clinical trial for POI, a disease associated with acute inflammation of the muscularis externa may be possible since it is available as a nutritional supplement for the relief of intestinal symptoms of IBD. One caveat is that PEA also activates other receptors in the gut and a more selective PPARα agonist would be preferred (Esposito et al., 2014).

### Endothelin-B Receptor Signaling in Glia

Our recent work supports the novel hypothesis that glial endothelin-1 (ET-1)/endothelin-B receptor (ET_B_R) signaling in enteric glia disrupts motility and it contributes to the pathogenesis of POI and POGD in the context of intestinal inflammation (Christofi et al., 2018; Mazzotta et al., 2019).

Pathogenic mechanism(s) under investigation include glial hypersensitivity to ET-1/ET_B_R signaling, induction of a reactive glial phenotype, enteric gliosis, enteric neuropathy and ENS dysfunction, and specific alterations in neural-motor pathways. Ongoing studies in our laboratory supported by the National Institute of Diabetes, Digestive and Kidney Diseases (NIDDK) will investigate whether the glial ET-1/ET_B_R signaling pathway is a potential novel therapeutic target in the prophylaxis against deleterious effects in glia, neurons and motility in POI and POGD.

Ongoing studies in our laboratory supported by NIH R01 DK113943 and R01 DK125809 are aimed to expand our current understanding of the pathogenic mechanisms in reactive glia linked to POI. Pathogenic targets of investigation include ATP purinergic signaling [or related nucleotides such as UTP, UDP, ADP] mediated through P2X or P2Y receptors on glia, endothelin-1/ET_B_ receptor signaling and IL1β/IL1R Signaling.

## Does Postoperative Gastrointestinal Tract Dysfunction Impact Long-Term Functional Recovery After Surgery and Quality of Life in the Patient?

Little is known about quality and functional recovery after surgery (Feldman et al., 2015). It is not clear if POGD has a downstream effect on long-term recovery and quality of life. The enteric nervous system, often called “the little brain,” has similarities with the central nervous system “the big brain.” It is possible to draw some parallels between neuroinflammatory disorders that affect these two organs, specifically POGD and POCD (postoperative cognitive dysfunction). For instance, we know that POCD is an important complication after surgery, especially in the elderly patient, with short term and long-term complications that significantly impact their quality of life (Steinmetz et al., 2009; Rundshagen, 2014). It is also possible and quite probable that POGD may represent short- and long-term complications during the acute phase involving inflammation, and long term after inflammation has resolved and the patient leaves the hospital. A recent study in neurogastroenterology and motility provided proof of concept, by showing for the first time that POI significantly decreases the quality of life at 3 and 6 months (QoL) (Peters et al., 2020). Therefore, it would be prudent to incorporate quality of life questionnaires in the CERAS protocols and extending the timeline to a longer postoperative period to uncover potential complications occurring after intestinal or systemic inflammation has resolved to further investigate long-term outcomes and mechanisms in POGD.

## Colorectal Enchanced Recovery After Surgery Protocols

ERAS guidelines are evidence-based protocols designed to standardize medical care, accelerate patient recovery, attenuate surgical stress response, improve patients’ outcomes and reduce the length of stay (LOS) and associated costs (Kehlet and Wilmore, 2008). ERAS involves a holistic, multimodal and articulate approach involving perioperative care.

Most of the Colorectal ERAS (CERAS) protocols combine 15–20 variables and a multidisciplinary group is required to coordinate each phase of the perioperative periods (Kehlet and Wilmore, 2008). Even though the relative contribution of each element to the utmost outcomes has not been determined, reduced stress response and accelerated recovery have been consistently reported in patients undergoing surgery within ERAS protocols (Varadhan et al., 2010; Kehlet and Joshi, 2017; Slim and Joris, 2017). In spite of the favorable perioperative outcomes reported in the first CERAS protocol in 2005, these guidelines were not widely implemented until recent years (Greco et al., 2014; Miller et al., 2014; Bakker et al., 2015; Nelson et al., 2016; Kehlet and Joshi, 2017; Pecorelli et al., 2017).

### Laparoscopic Surgery

Minimally invasive techniques are the cornerstone of ERAS protocols. Initial pre-clinical and clinical trials showed an association between smaller incisions and minimal or gentle gut manipulation with reduced surgical trauma, inflammation, and GI dysfunction (Böhm et al., 1995; Kehlet, 1999; Novitsky et al., 2004; Mamidanna et al., 2012). A faster resolution of GI dysfunction and reduced length of hospital stay have been reported in patients undergoing laparoscopic surgery when compared to conventional open surgical approaches (Schwenk et al., 1998; Schwenk et al., 2005; Van Bree et al., 2011; Mazzotta et al., 2020). However, when the open surgical approach is combined with ERAS protocols, POI incidence may be comparable between open and laparoscopic groups (Lei et al., 2015). Nevertheless, a recent report suggests that in the context of ERAS, laparoscopic techniques are associated with better immunologic response and shorter duration of POI (Wang et al., 2018).

### Early Feeding and Nasogastric Tube

Nasogastric (NG) tube insertion along with liberal parenteral hydration were routinely indicated during the postoperative period of GI surgery. Traditionally, the common practice involved leaving the GI tract to rest after surgery expecting faster healing. Therefore, the return of GI function was mandatory before the resumption of enteric nutrition (Verma and Nelson, 2007). However, little evidence supported these methods. In contrast, a growing body of evidence suggested that early feeding was associated with a significant reduction in postoperative complications and length of hospital stay (Lewis et al., 2001; Andersen et al., 2006; Lewis et al., 2009; Zhuang et al., 2013).

### Multimodal Analgesia

Postoperative pain management after abdominal surgery may be challenging for health care providers. Moreover, early pain control and GI functional recovery are both essential in CERAS protocols. Despite being effective analgesics, reduced GI motility is commonly reported in patients receiving opioids for postoperative pain management (Viscusi et al., 2009).

Multimodal analgesia combines regional analgesia, non-opioid analgesics [acetaminophen, nonsteroidal anti-inflammatory drug (NSAID) or cyclooxygenase (COX)-2 specific inhibitor], lidocaine infusions, gabapentinoids and ketamine. Numerous studies have shown the opioid-sparing effect of this approach resulted in an accelerated GI recovery and improved outcomes. However, an optimal combination of these elements has not yet been elucidated (Geltzeiler et al., 2014; Miller et al., 2014; Helander et al., 2017; Wick et al., 2017).

### Regional Analgesia

#### Thoracic Epidural

Thoracic epidural accelerates peristalsis by blocking pain afferents and efferent sympathetic inhibitory nerves. Epidural blocks are commonly used in patients undergoing open and complex abdominal surgeries (Gustafsson et al., 2012). In 2016, a Cochrane review of patients undergoing abdominal surgery reported a relevant association between epidural analgesia with an accelerated return of flatus and bowel movements (Khan et al., 2013; Guay et al., 2016). However, some reports suggested that this technique may also increase LOS after laparoscopic abdominal procedures under ERAS protocols (Nimmo and Harrington, 2014; Hübner et al., 2015; Borzellino et al., 2016).

#### Transverse Abdominis Plane Block

Transverse abdominis plane block (TAP block) is an effective alternative in patients undergoing abdominal surgeries. Torgeson et al. (2018) studied the non-analgesic outcomes in 78 patients undergoing open and laparoscopic colorectal surgery under general anesthesia, receiving either a TAP or an epidural block. A significant reduction in LOS (2.8 vs. 3.3 days, *p* = 0.026) was reported in the TAP block group with comparable results in GI recovery variables. Of note, the incidence of postoperative nausea and vomiting was higher in the TAP block group (31.7 vs. 13.5%; *p* = 0.057) (Torgeson et al., 2018).

### Nonsteroidal Anti-Inflammatory Drugs and Acetaminophen

Nonsteroidal anti-inflammatory drugs (NSAIDs) such as ketorolac and ibuprofen, mainly block the cyclooxygenase (COX) enzyme inhibiting the prostaglandin biosynthesis with subsequent decreased pain receptor activation. Similarly, an opioid-sparing effect and decreased opioid-related side effects have been reported after NSAIDs use (Elia et al., 2005; Straube et al., 2005).

Pre-clinical data suggests that eicosanoids play a key role in the development of POI, with NSAIDs being potentially able to accelerate GI recovery (Cheng et al., 1996; Kalff et al., 1998). However, NSAIDs’ analgesic ceiling and clinically relevant side effects (i.e., platelets dysfunction, GI bleeding, and renal dysfunction) may limit their use in patients with GI tract pathologies (Strom et al., 1996). Moreover, selective COX-2 inhibitors have a potent analgesic and anti-inflammatory effect but, its use in this patient setting has been associated with higher rates of anastomotic leak (Klein et al., 2011; Rushfeldt et al., 2011). In contrast, acetaminophen may be an acceptable alternative therapy for pain management (Apfel et al., 2013). Oral, intravenous and rectal formulations are available.

### Adjuvant Analgesic Medications

Lidocaine infusion is commonly known for its anti-inflammatory properties and opioid-sparing effect (Knotkova and Pappagallo, 2007; Dunn and Durieux, 2017). In addition to reduced postoperative pain scores, lidocaine infusion may improve GI recovery and shorten LOS after open abdominal surgeries (Sun et al., 2012). Other adjuvant agents such as gabapentin and ketamine, are widely known by their anti-hyperalgesic effect and a subsequent reduction in opioid consumption that potentially decreases the incidence and duration of POGD (Bell et al., 2006; Hurley et al., 2006; Mishriky et al., 2015).

### Peripherally Acting μ-Opioid Receptor Antagonist (PAM-OR)

Autonomic GI dysfunction is a well-known side effect of µ-receptor agonists. Opioids’ effects on central and intestinal µ-receptors may result in impaired GI motility. Some pharmacologic agents decrease the incidence and duration of POI by selectively blocking the intestinal (i.e., peripheral) µ-receptor (Sobczak et al., 2014). Alvimopan, an oral PAM-OR, has been approved by the Food and Drug Administration (FDA) for more than a decade to prevent opioid-induced constipation and POI. There is an important body of evidence indicating that Alvimopan enhances GI recovery in patients who received high doses of opioids after open abdominal surgery (Delaney et al., 2007; Vaughan-Shaw et al., 2012). However, in light of ERAS protocols involving reduced opioids requirement, the usefulness of Alvimopan in minimally-invasive colorectal surgery has been recently questioned (Keller et al., 2016).

### Bowel Preparation

Mechanical bowel preparation with oral antibiotics (MBP-OAB) is commonly indicated to prevent surgical site infection (SSI), anastomotic leak and ileus after elective colorectal surgery (Kiran et al., 2015; Morris et al., 2015; Holubar et al., 2017). However, a recent randomized clinical trial by Koskenvuo et al. (2019) reported that MBP-AOB does not reduce SSI or the overall morbidity (including POI) when compared to no bowel preparation (NBP) in patients undergoing elective colonic surgery.

### Perioperative Fluid Management

The gut is highly susceptible to interstitial edema. Excessive perioperative fluid administration may lead to edema contributing to POI and delay in GI recovery, delay in the gut’s anastomotic healing and delay in hospital discharge (Gan et al., 2002; Holte et al., 2002; Lobo et al., 2002; Moore-Olufemi et al., 2005; Nisanevich et al., 2005; Uray et al., 2006; Schnüriger et al., 2011). However, in the context of CERAS programs with more conservative fluid therapies, the effects of edema may be importantly attenuated (Srinivasa et al., 2013; Gómez-Izquierdo et al., 2015; Gómez-Izquierdo et al., 2017).

### Supportive Treatment and Symptom Control

Supportive treatment and symptom control are paramount during POGD management. The first step is to rule out any acute intra-abdominal condition or other surgical complications. Serial radiographic imaging and computed tomography (CT) scan should be considered (Sandrasegaran and Maglinte, 2005). Supportive care may include the removal of any potential triggers such as opioids and fluid overload. Moreover, bowel rest with NG tube insertion may be considered for gastric decompression and pulmonary aspiration risk (Adiamah and Lobo, 2020).

## Current Therapies Listed in Clinical Trials.gov

A search of Clinical Trials.gov identified 125 ongoing clinical trials, with many in advanced clinical trials (Phase III or Phase IV trials), including new or old drugs, herbal medications, acupuncture and Vagus Nerve Stimulation (VNS). Methylnaltrexone and Naldemedine are peripheral acting μ‐opioid antagonists to prevent opioid induced slow transit constipation. Simethiocone is in a Phase IV clinical trial for POI that acts as an anti-bloating and anti-flatulence medication by reducing the surface tension of the gas. Ulimovelin is a ghrelin agonist being tested as a prokinetic agent in a new Phase III clinical trial. Ulimorelin was shown to be ineffective in preventing POI in an earlier large RCT (Shaw et al., 2013). Historically, prokinetic agents have been commonly used to treat POI. However, a large body of evidence has shown little or no benefits after administrating neostigmine, erythromycin, or metoclopramide in patients with impaired GI function (Myrhöj et al., 1988; Tollesson et al., 1991; Smith et al., 2000). Lidocaine infusion is in a Phase II clinical trial for prophylaxis of POGD for radical systectomy. Beet root juice and TU-100 are examples of herbal medications in Phase II trials for POI and POGD. Drinking coffee and nicotine chewing gum are other approaches under investigation. Chewing gum was traditionally known to expedite GI recovery after abdominal surgery (Li et al., 2013). However, most of the studies supporting this effect were published before the implementation of early postoperative feeding as part of the ERAS protocols (Ho et al., 2014). The effects of chewing gum are described in a systematic review (Short et al., 2015).

Caffeine is widely known as a stimulant to colonic motor activity in animals and humans (Rao et al., 1998). Clinical trials have shown that caffeinated drinks decrease the time to flatus and first bowel movement and if given as soon as 2 h after surgery, it may accelerate GI recovery and reduce LOS (Müller et al., 2012; Dulskas et al., 2015; Göymen et al., 2017; Kane et al., 2020).

## Conclusion

Our review focused on recent advances and understanding in pathogenic mechanisms, treatment strategies, pipeline drugs and ongoing clinical trials and approved medications for those targets for POI and POGD. Further clinical studies on 5HT_4_R agonists and vagal nerve stimulation are required to establish their usefulness as novel therapies, and more work needs to be done of short and long term impact of gut surgical manipulation on patient outcomes. A key target of investigation should be to pinpoint the triggering mechanism(s) in various cells in the intestinal wall, and better understand the dynamic interactions between various cells implicated in the disease. Enteric glial cells (EGCs) are abundant in the gut and they may play a role in the pathophysiology of POI and POGD. Our review provides some examples of how targeting different cells in the gut wall, can potentially identify novel therapeutic targets for POI and POGD. Despite the implementation of enhanced recovery protocols for GI surgeries, there is still significant POI and POGD associated with prolonged hospitalizations, and increased morbidity and healthcare costs. We cannot deny that significant progress has been made with ERAS protocols and other advances in the field to reduce the incidence and overall morbidity associated with this iatrogenic disorder. Novel therapies in the pipeline offer some hope for better treatments, but a better understanding of the pathogenic mechanism(s) of POI is required to develop better therapeutic strategies (Collins et al., 2016). As food for thought, the NIH recently published its strategic plan. In that document, NIH reaffirmed its strong commitment for basic scientific discovery noting that many of the most important medical advances trace back to basic research, which had no explicit link [NIH, NIH-Wide Strategic Plan (www.nih.gov/about-nih/nih-wide-strategic-plan)]. Further research into basic mechanisms of immune/inflammatory cells (muscularis macrophages, dendritic cells, leukocytes, monocytes, mast cells), “reactive enteric glia”, ICC’s, intrinsic and extrinsic neural pathways, and the microbiome in the gut lumen is an essential step in developing novel treatment strategies, but a big hurdle is translatability of findings in animal models to humans. This will allow us to improve our CERAS protocols in the prophylaxis against POI and POGD.

## Abbreviations

**Abbreviations:** ATP, adenosine-5′-triphosphate; CERAS, colorectal enhanced recovery after surgery; ET-1, endothelin-1; ETBR, endothelin-B receptor; GI, gastrointestinal; NG, nasogastric; POGD, postoperative gastrointestinal tract dysfunction; POI, postoperative ileus; 5-HT, 5-hydroxytryptamine (serotonin); IL-1β, interleukin-1β; IL-6, interleukin-6; IL-8, interleukin-8; IL-1R, interleukin-1 receptor; VNS, vagus nerve stimulation
